# Microarray Analysis of Rice d1 (RGA1) Mutant Reveals the Potential Role of G-Protein Alpha Subunit in Regulating Multiple Abiotic Stresses Such as Drought, Salinity, Heat, and Cold

**DOI:** 10.3389/fpls.2016.00011

**Published:** 2016-01-28

**Authors:** Annie P. Jangam, Ravi R. Pathak, Nandula Raghuram

**Affiliations:** University School of Biotechnology, Guru Gobind Singh Indraprastha UniversityDwarka, India

**Keywords:** G-protein, heat, cold, salt, drought, stress, rice, RGA1

## Abstract

The genome-wide role of heterotrimeric G-proteins in abiotic stress response in rice has not been examined from a functional genomics perspective, despite the availability of mutants and evidences involving individual genes/processes/stresses. Our rice whole transcriptome microarray analysis (GSE 20925 at NCBI GEO) using the G-alpha subunit (RGA1) null mutant (Daikoku 1 or d1) and its corresponding wild type (*Oryza sativa* Japonica Nipponbare) identified 2270 unique differentially expressed genes (DEGs). Out of them, we mined for all the potentially abiotic stress-responsive genes using Gene Ontology terms, STIFDB2.0 and Rice DB. The first two approaches produced smaller subsets of the 1886 genes found at Rice DB. The GO approach revealed similar regulation of several families of stress-responsive genes in RGA1 mutant. The Genevestigator analysis of the stress-responsive subset of the RGA1-regulated genes from STIFDB revealed cold and drought-responsive clusters. Meta data analysis at Rice DB revealed large stress-response categories such as cold (878 up/810 down), drought (882 up/837 down), heat (913 up/777 down), and salt stress (889 up/841 down). One thousand four hundred ninety-eight of them are common to all the four abiotic stresses, followed by fewer genes common to smaller groups of stresses. The RGA1-regulated genes that uniquely respond to individual stresses include 111 in heat stress, eight each in cold only and drought only stresses, and two genes in salt stress only. The common DEGs (1498) belong to pathways such as the synthesis of polyamine, glycine-betaine, proline, and trehalose. Some of the common DEGs belong to abiotic stress signaling pathways such as calcium-dependent pathway, ABA independent and dependent pathway, and MAP kinase pathway in the RGA1 mutant. Gene ontology of the common stress responsive DEGs revealed 62 unique molecular functions such as transporters, enzyme regulators, transferases, hydrolases, carbon and protein metabolism, binding to nucleotides, carbohydrates, receptors and lipids, morphogenesis, flower development, and cell homeostasis. We also mined 63 miRNAs that bind to the stress responsive transcripts identified in this study, indicating their post-transcriptional regulation. Overall, these results indicate the potentially extensive role of RGA1 in the regulation of multiple abiotic stresses in rice for further validation.

## Introduction

Abiotic stress responses in plants are being increasingly addressed on a genome-wide scale to find newer gene targets for protecting crop yields in the era of climate change (Pandey et al., 2015). Rice has been a crop of particular interest in this regard, not only because of its popularity as a post-genomic model crop, but also its importance as a staple food for half of the world's population. In rice, transcriptome-wide analyses of abiotic stress response have been reported in terms of either specific stresses, or specific families of genes that respond to multiple stresses, or both. They include drought-responsive (Wang et al., 2011) and salinity-responsive (Jiang et al., 2013) rice transcriptomes spanning multiple gene families, pathways, and transcription factors. Studies that examined multiple stresses in parallel include transcriptome-wide response to water-deficit, cold, and salt stress in rice (Ray et al., 2011; Venu et al., 2013).

There have been many other whole transcriptome microarray studies in rice under different abiotic stress conditions, but they reported only specific gene families that responded to various stresses. They include the MADS-box transcription factor family (Arora et al., 2007), F-Box Proteins (Jain et al., 2007), calcium-dependent protein kinase (CDPK) gene family (Ray et al., 2007), auxin-responsive genes (Jain and Khurana, 2009), protein phosphatase gene family (Singh et al., 2010), Sulfotransferase (SOT) gene family (Chen et al., 2012), thioredoxin gene family (Nuruzzaman et al., 2012), half-size ABC protein subgroup G (Matsuda et al., 2012), class III aminotransferase gene family (Sun et al., 2013), Ca^2+^ ATPases gene family (Kamrul Huda et al., 2013), Rice RING E3 Ligase Family (Lim et al., 2013) etc.

Hetetrotrimeric G-protein signaling components have often been implicated in stress response in plants. For example, in pea, Gα subunit was shown to be up-regulated by heat, as well as to impart heat and salt tolerance when overexpressed in transgenic tobacco, whereas the Gβ subunit imparted only heat tolerance (Misra et al., 2007). The role of α subunit in salt stress has also been shown in Arabidopsis (Colaneri et al., 2014), rice, and maize (Urano et al., 2014). Recently, we demonstrated that stress-related genes/pathways constitute the largest functional cluster of GPCR/G-protein-regulated genes in *Arabidopsis* using whole transcriptome analyses of knock-out mutants of GCR1 and GPA1 (Chakraborty et al., 2015a,b).

The rice G protein subunits are well characterized as RGA1 for Gα subunit (Ishikawa et al., 1995), RGB1 for Gβ subunit (Ishikawa et al., 1996) and RGG1 and RGG2 for the Gγ subunits (Kato et al., 2004). The expression of rice Gα subunit (RGA1) gene was reported to be up-regulated by salt, cold, and drought stresses, and down regulated by heat stress (Yadav et al., 2013). However, the regulation of the two Gγ subunits was different—while both RGG1 and RGG2 were up-regulated in salt, cold, heat, and ABA treatments, only RGG1 was up-regulated in drought stress (Yadav et al., 2012). While these two studies demonstrated that abiotic stresses regulate the expression of Gα and Gγ genes in rice, the role of G-proteins in mediating various stress responses in rice remains uncharacterized on a genome-wide scale. The availability of a natural mutant of RGA1 (D1) in rice (Ashikari et al., 1999) makes a functional genomic approach particularly attractive in this regard. We carried out a microarray analysis of this RGA1 mutant in comparison with the wild type in rice (GSE 20925 at NCBI GEO), which provided a convenient starting point for the present study, to examine the stress-related genes in the genome-wide response to the RGA1 null mutation in rice. In specific terms, we asked what proportion of the RGA1-regulated transcriptome corresponds to abiotic stress response in rice and how are these genes distributed in terms of major individual abiotic-stresses or in terms of their differential regulation in the RGA1 mutant or normal rice plants. We report here an integrative analysis of our experimental RGA1 mutant microarray data with the *in silico* meta data analysis of the known response of normal rice plants to various abiotic stresses.

## Materials and methods

### Plant material and growth conditions

Seeds of the rice d1 mutant (devoid of Gα subunit or RGA1) and its corresponding wild type (*Oryza sativa* japonica Nipponbare) were obtained from the Faculty of Agriculture, Kyushu University, Japan. They were surface-sterilized with 70% ethanol and 0.01% Triton-X 100 and grown on 0.5x B5 media containing 0.7% agar at 25 ± 1°C with fluorescent white light intensity of 1 kilo lux and a 12/12 photoperiod for 25 days till the emergence of the tertiary leaves and used for microarray analysis.

### RNA isolation and analysis

Total RNA was isolated by hot phenol extraction and lithium chloride precipitation method as described (Pathak and Lochab, 2010). Total RNA was qualitatively and quantitatively analyzed by spectrophotometry and agarose gel electrophoresis. Prior to microarray experiments, RNA integrity values (RIN) of the total RNA samples were determined using the Agilent 2100 Bionalyzer equipment as per the manufacturer's instructions and only samples with RIN values higher than 5 were used for microarray experiments.

### Whole transcriptome microarray

cRNA labeling of total RNAs from the RGA1 mutant and its corresponding wild type was carried out using Agilent Low RNA Input Fluorescent Linear Amplification Kit (USA) as per the manufacturer's instructions, using Cy3 and Cy5 dyes (Perkin-Elmer, USA). Amplified samples were purified using Qiagen's RNeasy mini spin columns. The quantity and specific activity of cRNA was determined by using NanoDrop ND-1000 Spectrophotometer. Samples with specific activity >8 were hybridized with Agilent rice whole genome 60-mer microarrays (4 × 44 K, Ver 2) at 65°C for 17 h using Agilent Microarray Hybridization materials and equipment, as per the manufacturer's instructions. Slides were washed for 1 min each with Agilent Gene expression Wash Buffer I and II at RT and 37°C, respectively, and rinsed with acetonitrile for cleaning up and drying. They were scanned on an Agilent scanner (G2565B) at 100% laser power. Data extraction was carried out with Agilent Feature Extraction software (version 9.1).

The raw data was normalized using the recommended “Per Chip and Per Gene Normalization” feature of the software GeneSpring GX Version 11.5. The correlation of replicates was checked using principal component analysis and correlation coefficients were obtained. The geometric mean (geomean) fold change values are represented as log_2_. The average data of biological replicates were used for final calculations. Log_2_ fold change value of 1.0 with a *p*-value of 0.05 was taken as the cut-off to identify the differentially regulated genes (DEGs).

### Data mining and meta-analysis of the stress related genes

The stress-related genes were segregated from the above RGA1-regulated DEGs using the GO term “stress.” This was done using rice genome annotation version 7 and also validated with the “manually curated database for rice proteins” (Gour et al., 2013). Further data mining was done using the genes corresponding to individual stresses downloaded from the stress responsive transcription factor database (STIFDB2.0, Naika et al., 2013), to find RGA1-regulated DEGs corresponding to heat, drought, salt, and cold. In order to identify additional stress-related genes among RGA1-responsive genes, our entire RGA1-regulated transcriptome was used as an input at the online database RiceDB (Narsai et al., 2013) to identify all the rice genes that responded to at least one of the four abiotic stresses i.e., cold, heat, drought, and salt. These genes were sorted into up-regulated and down-regulated sets and subjected to various Venn selections (Oliveros, 2007–2015) to generate a core list of 1498 stress-responsive genes common to all four stresses in rice. The core gene list was further classified into various functional categories, pathways and processes using a GO enrichment analysis tool, AGRIGO (Du et al., 2010) with binomial statistical test and cut-off for FDR-adjusted *P*-value of 0.05. Hierarchical clustering was done using average linkage based on Euclidean distance subsets of individual stress conditions such as heat, cold, drought/dehydration, salt, submergence, and shift from aerobic to anaerobic germination, cold, and drought. Biclustering was done with a threshold value of 1 and the largest bicluster was used for the analysis. Expression data were obtained for both the clustering analyses using Genevestigator (Zimmermann et al., 2004).

### RT-PCR validation of the stress-related genes

In order to validate the stress-responsive genes identified from the microarray results, quantitative RT-PCR experiments were carried out using total RNAs isolated from two biological replicates of the wild type and RGA1 mutant rice plants grown and harvested under similar conditions. Two technical replicates were used to set up RT-PCR from each of the biological replicates, using gene-specific primers designed in-house for the selected genes. The primer sequences are provided in Supplementary Table 2. PCR amplifications were performed in 20 μl by using the KAPA SYBR FAST universal QPCR kit (KAPA BIOSYSTEMS) with 1.0 μl of sample cDNA prepared by using iScript cDNA synthesis kit (Cat#170-8891) from BIORAD and 100 n moles of each gene-specific primer. Actin (ACT) was used as an internal control for normalization. Quantification of the relative changes in gene expression was performed by using the 2^−ΔΔCT^ method (Pfaffl, 2001).

## Results

Whole transcriptome microarray analysis of the rice RGA1 (Gα) null mutant in comparison with its WT yielded a total of 2270 differentially expressed genes under MIAME compliant conditions, using stringent cut-off values (geomean 1.0 with *p*-value of 0.05) and removing redundancies. The raw data of this entire microarray experiment are reported at NCBI GEO (GSE 20925). Among these RGA1-regulated genes, a large number of abiotic stress-responsive genes have been identified using their annotation information or online databases for further bioinformatic analysis as detailed below.

### Stress-responsive genes identified by GO-terms

Our search for stress-related genes among these RGA1-regulated DEGs using the GO terms related to stress yielded 94 abiotic stress-related DEGs that are nearly equally distributed in terms of up/down regulation (49 up/45 down). A vast majority of these genes could be clustered into < 40 related families (20 up/20 down) showing identical mode of up/down regulation, despite wide variation in the extent of their regulation (Table 1). For example, all the RGA1-regulated members of gene families such as DREB seem to be uniformly up-regulated, albeit to varying extents, ranging between +3.99 and +1.18. In addition, there are 21 stress-related DEGs that are individually regulated in the RGA1 mutant with no other family member, including up-regulated genes such as CDPK, MAP kinase kinase 2, DnaJ like protein, and down-regulated genes such as Myb factor, phytochelatin synthetase, and water-stress inducible protein (RAB21).

**Table 1:** **RGA1-regulated stress-responsive gene families with their fold changes in mutant**.

**RGA1 up regulated stress responsive families**	**Fold change**	**RGA1 down regulated stress responsive families**	**Fold change**
DRE-binding protein 1A,1B,1C	3.99–1.18	Peroxidase	−6.09 to −1.42
Metallothionein-like protein type 2	3.88–3.47	Catalase isozyme	−3.33 to −3.09
Catalase isozyme 2	2.89–2.6	Haem peroxidase family protein.	−2.88 to −1.47
Glutathione S-transferase GST 19,6	2.49–1.55	LRR-like protein	−2.71 to −2.54
Heat shock proteins (70,82,90)	2.31–1.47	Pathogen-inducible alpha dioxygenase	−2.60 to −2.58
MAP Kinase	2.19–2.06	Triosephosphate isomerase	−1.89 to −1.63
Endo-1, 3; 1, 4-beta-D-glucanase	2.06–1.55	Anth (Pollen-specific desiccation-associated LLA23 protein)	−1.86 to −1.59
Rossmann-like alpha/beta/alpha sandwich fold domain containing protein	2.05–1.28	Cytosolic 6-phosphogluconate dehydrogenase	−1.81 to −1.72
Zinc finger, domain containing proteins (AN1-type, TAZ-type)	2.04–1.50	Glycine-rich RNA-binding protein 1	−1.79 to −1.72
Heat stress transcription factor Spl7 (RHSF10)	1.99–1.11	Beta-glucanase precursor	−1.72 to −1.64
Sucrose synthase 2	1.84–1.33	Cytosolic Glyceraldehyde-3-phosphate dehydrogenase	−1.70 to −1.50
Anthranilate synthase beta chain	1.75–1.44	Superoxide dismutase	−1.69 to −1.49
Manganese-superoxide dismutase precursor	1.52–1.26	WW/Rsp5/WWP domain containing protein	−1.59 to −1.33
6-phosphogluconate dehydrogenase	1.46–1.35	Serine/threonine-protein kinase SAPK3 (Osmotic stress/abscisic acid-activated protein kinase 3)	−1.46 to −1.36
Serine/threonine-protein kinase SAPK2/9	1.34–1.05	Alcohol dehydrogenase 1.	−1.42 to −1.23
UspA domain containing protein	1.30–1.18	Class III peroxidase GvPx2b	−1.39 to −1.08
GTP-binding nuclear protein Ran1B	1.15–1.04	Nucleoside diphosphate kinase I	−1.36 to −1. 33
Multiple stress-responsive zinc-finger protein ISAP1 (Stress-associated protein 1)	1.14–1.09	Hypothetical conserved gene. Similar to peroxidase (Os03t0339300-02)	−1.25 to −1. 07
OSIGBa0145M07.4 protein	1.83–1.14	Aldehyde dehydrogenase	−1.21 to −1.16

*From the differentially regulated genes (DEGs) identified in our microarray analysis of the RGA1 mutant, 94 genes with GO terms related to stress were segregated and categorized into families*.

### Stress-responsive genes identified at STIFDB2.0

**Figure 1:**
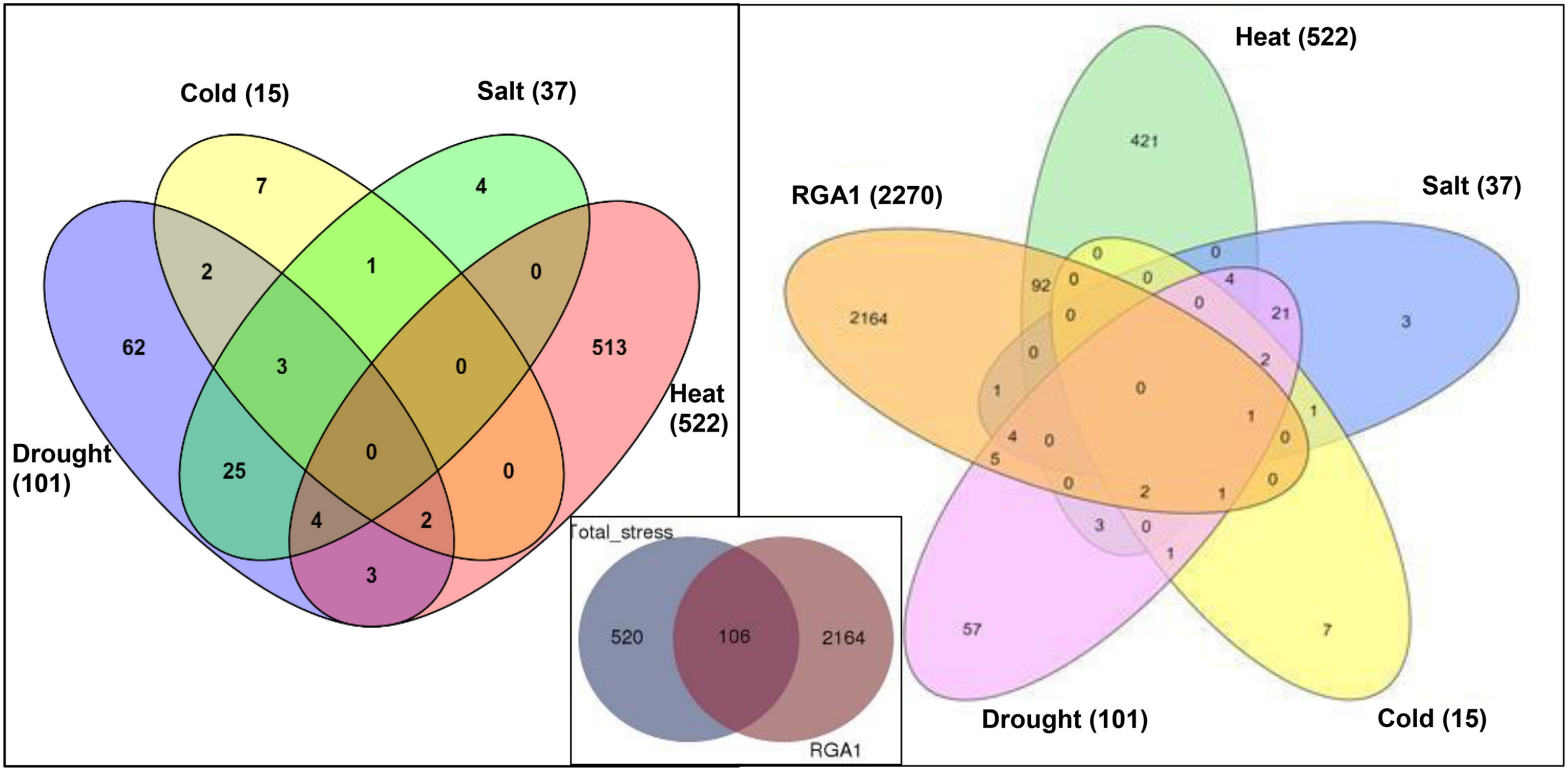
**Stress responsive genes among RGA1-regulated genes in rice**. The left panel shows Venn selections between the subsets of all rice abiotic stress-responsive genes listed at STIFDB2.0. The inset shows Venn selection between all 626 abiotic stress-responsive genes listed at STIFDB2.0 and 2270 RGA-1-regulated DEGs identified on our microarray. The left panel shows the break-up of the 106 RGA-regulated stress-responsive genes identified in the inset in terms of individual stresses viz., heat (94), drought (13), salt (6), and cold (4).

Data mining for all abiotic stress-responsive genes of rice at STIFDB2.0 yielded 626 genes in all, corresponding to heat (522), drought (101), salt (37), and cold (15), as shown in the left panel of Figure 1. A Venn selection between these 626 stress responsive genes and the 2270 RGA1-regulated genes identified on our microarray yielded 106 genes (Figure 1, inset), indicating the role of RGA1 in mediating their stress regulation. A significant majority of them respond to heat (94), followed by drought (13), salt (6), and cold (4), with the 25 genes being common to salt and drought stresses (Figure 1, right panel). But this order becomes very different when seen in terms of what proportion of each of the stress responses was RGA1-regulated: With four out of all 15 cold-responsive genes listed at STIFDB2.0 being regulated by RGA1, cold-response has the highest proportion of genes under the regulation of RGA1 (27%), followed by heat (18%), salt (16%), and drought (13%).

### Expression profiles of RGA1-regulated stress-responsive genes

**Figure 2:**
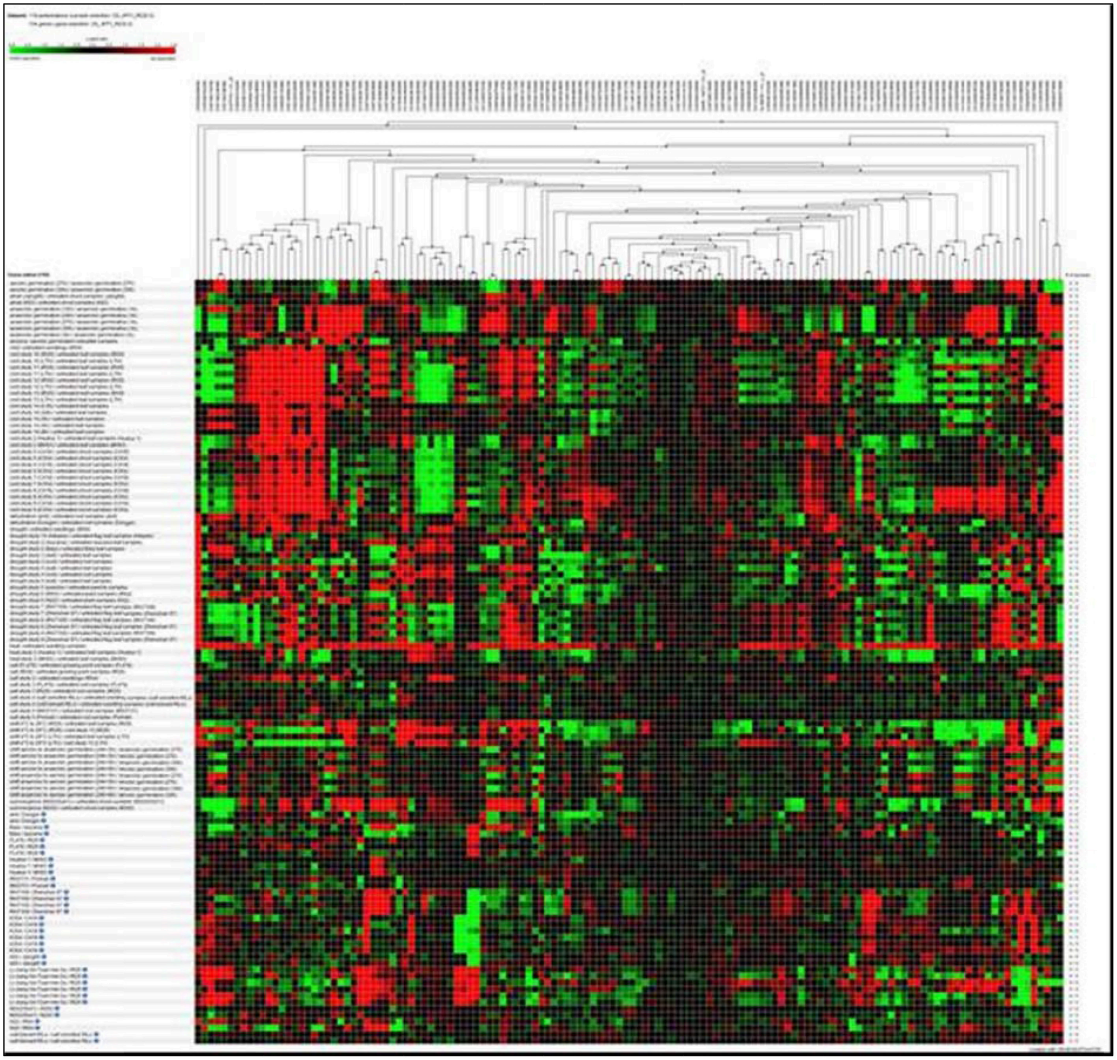
**Hierarchical clustering of the transcripts of 106 RGA1-regulated stress-responsive genes in various abiotic stress studies in rice (118 perturbations)**. The red and green colors indicate up-regulation (log2 [2.5]) and down-regulation (log2 [−2.5]), respectively as shown in the color bar. Hierarchical clustering was done using average linkage based on Euclidean distance subsets of individual stress conditions such as heat, cold, drought/dehydration, salt, submergence, and shift from aerobic to anaerobic germination cold and drought. The expression data were obtained using Genevestigator (Zimmermann et al., 2004).

Hierarchical clustering of the transcripts of the 106 RGA1-regulated stress responsive genes using Genevestigator revealed their differential expression under 132 perturbations related to abiotic stress studies reported in literature. Out of them, the data in Figure 2 include only 118 perturbations such as heat, cold, drought/dehydration, salt, submergence, and shift from aerobic to anaerobic germination, that have affected the expression of the vast majority of 106 genes queried based on our study. This revealed a prominent cluster of over 25 genes that are highly up-regulated (over 2.5-fold) and a similar number of highly down-regulated (over 2.5-fold) under 24 cold stress studies in literature on rice. There are an even larger number of genes that are differentially regulated under drought, of which the down-regulated genes are both predominant and better clustered, relative to the up-regulated genes. Though there are a smaller number of heat responsive genes, they are neither well clustered not consistent between different studies. With respect to salt, the results from nine studies show that very few of our 106 RGA-regulated genes respond to salt stress in rice. In view of these findings, further *in silico* analysis of transcript profiles was restricted to cold and drought stress conditions.

**Figure 3:**
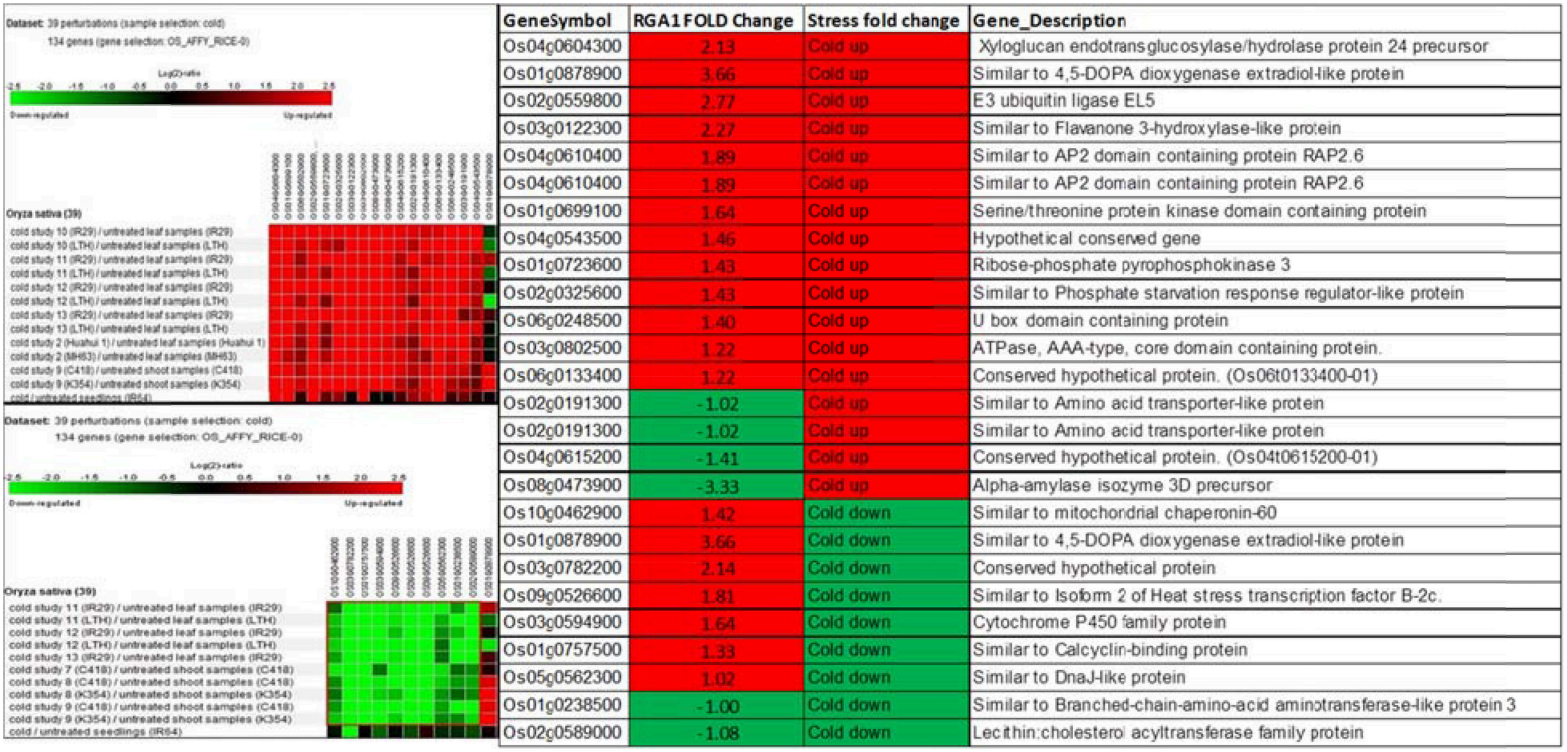
**Expression profiles of 106 RGA1-regulated stress-responsive genes in cold stress (39 perturbations from literature)**. The red and green colors indicate up-regulation (log2 [2.5]) and down-regulation (log2 [−2.5]), respectively, as shown in the color bar. The expression data in the left panel were obtained using Genevestigator. The table compares their regulation in normal plants under stress in literature with actual fold-change values in the RGA1 mutant.

Biclustering analysis of the expression profiles of 106 RGA1-regulated, stress-responsive genes in various studies on cold stress revealed that 17 genes were up-regulated and 11 genes were down-regulated in 39 different perturbations/studies (Figure 3, left panel). Their comparison with the actual fold-change values of those genes on our microarray revealed that about half of them are similarly up-regulated in both RGA1 mutant (without stress) as well as in normal rice plants under cold stress. The remaining genes include seven up-regulated genes and six down-regulated genes in the RGA1 mutant with opposite pattern of regulation under cold stress in normal rice plants in literature (Figure 3, right panel). The genes up-regulated in the RGA1 mutant but down-regulated by cold stress in the normal plants include mitochondrial chaperonin-60, 4,5-DOPA dioxygenase extradiol-like protein, isoform 2 of heat stress transcription factor B-2c, cytochrome P450 family protein, calcyclin-binding protein, DnaJ-like protein. The genes down-regulated in the RGA1 mutant but up-regulated in normal plants include amino acid transporter-like protein and alpha-amylase isozyme 3D precursor. The opposite pattern of regulation of these genes could be due to the RGA1 mutation, which indicates that RGA1 may mediate the response of these genes to cold stress.

**Figure 4:**
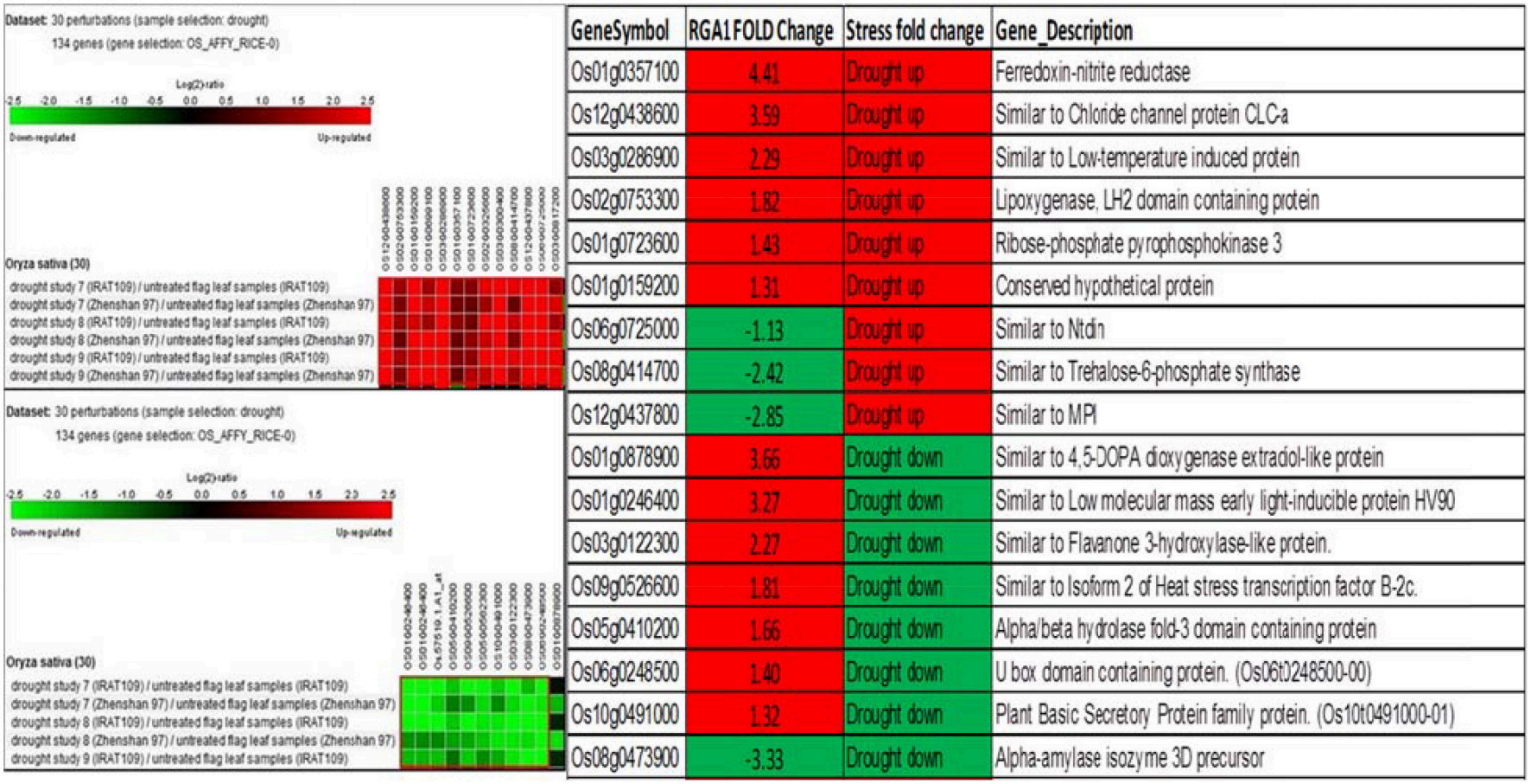
**Expression profiles of 106 RGA1-regulated stress- responsive genes in drought stress (30 perturbations from literature)**. The red and green colors indicate up-regulation (log2 [2.5]) and down-regulation (log2 [−2.5]), respectively, as shown in the color bar. The expression data in the left panel were obtained using Genevestigator. The table compares their regulation in normal plants under stress in literature with actual fold-change values in the RGA1 mutant.

A similar biclustering analysis of the expression patterns of 106 RGA1-regulated stress-responsive genes in studies on drought stress revealed that 13 genes were up-regulated and 10 genes were down-regulated in 30 different perturbations/studies (Figure 4, left panel). When their up/down regulation was compared with the actual fold-change values obtained on our microarray, six of the up-regulated genes and one of the down-regulated genes from literature are similarly up-regulated in both RGA1 mutant (without stress) as well as in normal rice plants under drought stress. Among the rest, seven up-regulated and three down-regulated genes in the RGA1 mutant showed opposite pattern of regulation under drought stress in normal rice plants in literature (Figure 4, right panel). The genes up-regulated in the RGA1 mutant but down-regulated by drought stress in the normal plants include flavanone 3-hydroxylase-like protein, Isoform 2 of heat stress transcription factor, B-2cAlpha/beta hydrolase fold-3 domain containing protein, U box domain containing protein, and plant basic secretory protein family protein. The genes down-regulated in the RGA1 mutant by up-regulated in normal plants include Trehalose-6-phosphate synthase, MPI, and Ntdin. The opposite pattern of regulation of these genes could be due to the RGA1 mutation, which indicates that RGA1 may mediate the response of these genes to drought stress.

Interestingly, ribose phosphate pyrophosphokinase 3 is up-regulated in the RGA1 mutant as well as in response to cold and drought stress in literature, whereas isoform 2 of the heat stress transcription factor is up-regulated in the RGA1 mutant, but down-regulated in drought and cold stresses.

### Meta-data analysis

**Figure 5:**
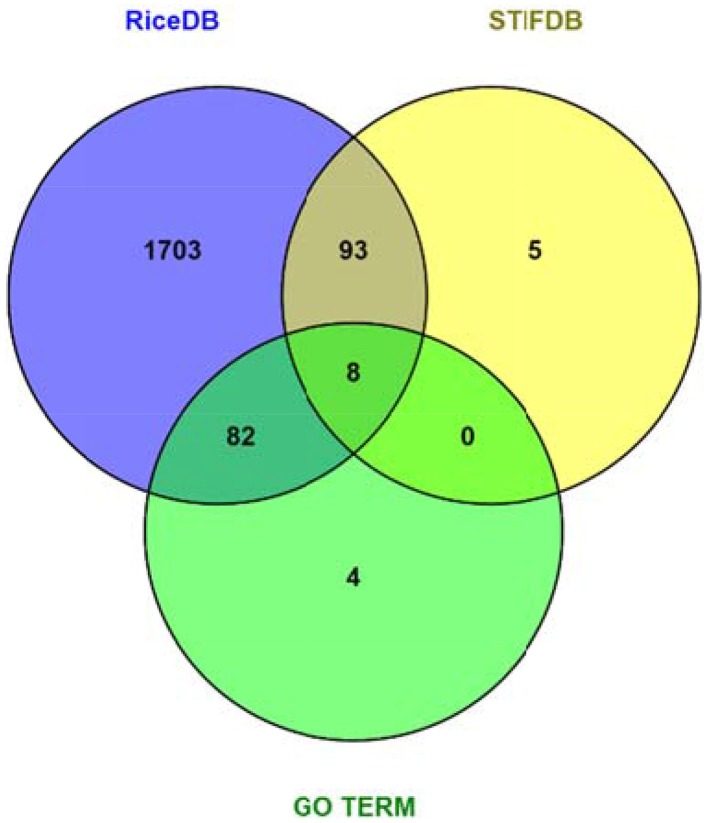
**Venn selection of RGA1 regulated stress responsive genes mined from RiceDB, STIFDB, and GO term**. The overlap among the three sets revealed that the genes mined using GO term stress and stress responsive genes from STIFDB are largely subsets of the 1886 DEGs identified using Rice DB.

Data mining using our entire non-redundant list of 2270 RGA1-regulated DEGs (1242 up and 1028 down) as input query at the Rice DB Oryza information portal revealed a much larger number of 1886 stress-related genes as differentially regulated in our RGA1 mutant. This prompted a comparison of various stress-responsive gene lists identified using different approaches in this study, such as gene ontology (94), STIFDB2.0 (106), and Rice DB (1886). A Venn selection of all three sets revealed that the former two are largely subsets of the 1886 DEGs identified using Rice DB (Figure 5). Therefore, the rest of the meta-data analysis was carried out using these 1886 genes.

**Figure 6:**
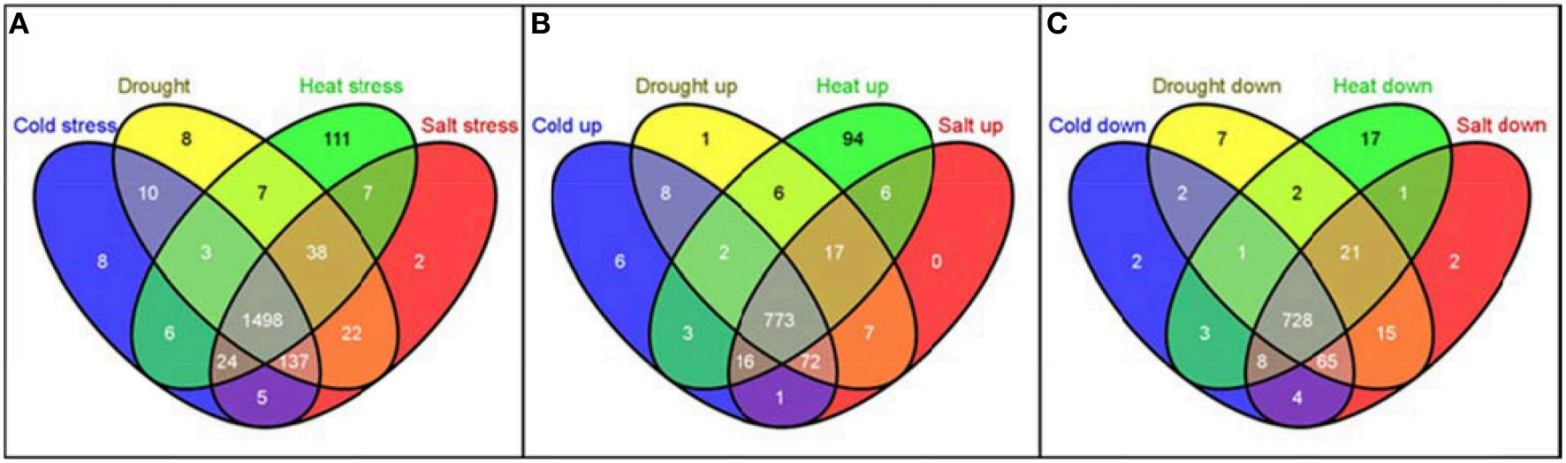
**Meta-data analysis of RGA1-regulated genes regulated under various abiotic stresses**. The 2270 RGA1-regulated genes (1242 up and 1028 down) were used as input query at Rice DB to generate genes responsive to cold (878 up/810 down), drought (882 up/837 down), heat (913 up/777 down), and salt stress (889 up/841 down) with 1498 genes common to all four stresses and totaling 1886 unique genes. Their Venn selections are depicted as total **(A)**, up-regulated **(B)** and down-regulated **(C)** sets, using the online tool Venny (Oliveros, 2007–2015).

The distribution of these 1886 RGA1-regulated, stress-responsive DEGs in terms of individual stresses was found to be 1730 DEGs in salt stress (889 up/841 down), 1719 DEGs in drought (882 up/837 down), 1690 DEGs in heat (913 up/777 down), and 1688 DEGs in cold (878 up/810) down with 1498 genes (773 up/725 down) common to all four stresses (Figure 6). In other words, as many as 1886 G-protein-regulated genes are responsive to one or more of these stresses, indicating their possible regulation through G-protein (RGA1) signaling.

Interestingly, the largest majority of 1886 with 1498 genes (or 80%) are common to all four abiotic stresses, followed by 137 genes common to cold, drought and salt stresses, followed by 38 genes common to drought, heat, and salt and so on, indicating their common regulation through G-proteins (Table 2). Even more interesting is the fact that as many as 111 heat-responsive genes are not common to any other stress and are uniquely regulated through G-proteins by heat only, followed by eight genes each in cold only and drought only, and two genes in salt stress only (Table 2). Some of the exclusively heat-responsive RGA1-regulated genes include superoxide dismutase, chitin-inducible gibberellin-responsive protein, brassinosteroid insensitive 1-associated receptor kinase 1, Hsp70 heat shock family protein, GTP-binding nuclear protein Ran1B (fragment), low affinity sulfate transporter 3, mitochondrial chaperonin-60, nucleoside diphosphate kinase I (EC 2.7.4.6) (NDPK I), wound responsive protein, and auxin response factor 2 (ARF1-binding protein).

**Table 2:** **Distribution of RGA1-regulated genes among major abiotic stresses in Rice DB**.

**Stress categories**	**Up regulated in RGA1 mutant**	**Down regulated in RGA1 mutant**	**Total**
Cold, drought, heat, and salt	773	728	1498^*^
Cold, drought, and salt	72	65	137
Drought, heat, and salt	17	21	38
Cold, heat, and salt	16	8	24
Drought and salt	7	15	22
Cold and drought	8	2	10
Drought and heat	6	2	8
Heat and salt	6	1	7
Cold and heat	3	3	6
Cold and salt	1	4	5
Cold, drought, and heat	2	1	3
Heat only	94	17	111
Cold only	6	2	8
Drought only	1	7	8
Salt only	0	2	2

*The 1886 RGA1-regulated genes identified as responsive to abiotic stresses at Rice DB have been categorized in terms of shared/unique stress categories and their up/down regulation in the RGA1 mutant*.

* *Three genes out of 1501 were redundant or common to up/down categories, hence 1498*.

**Figure 7:**
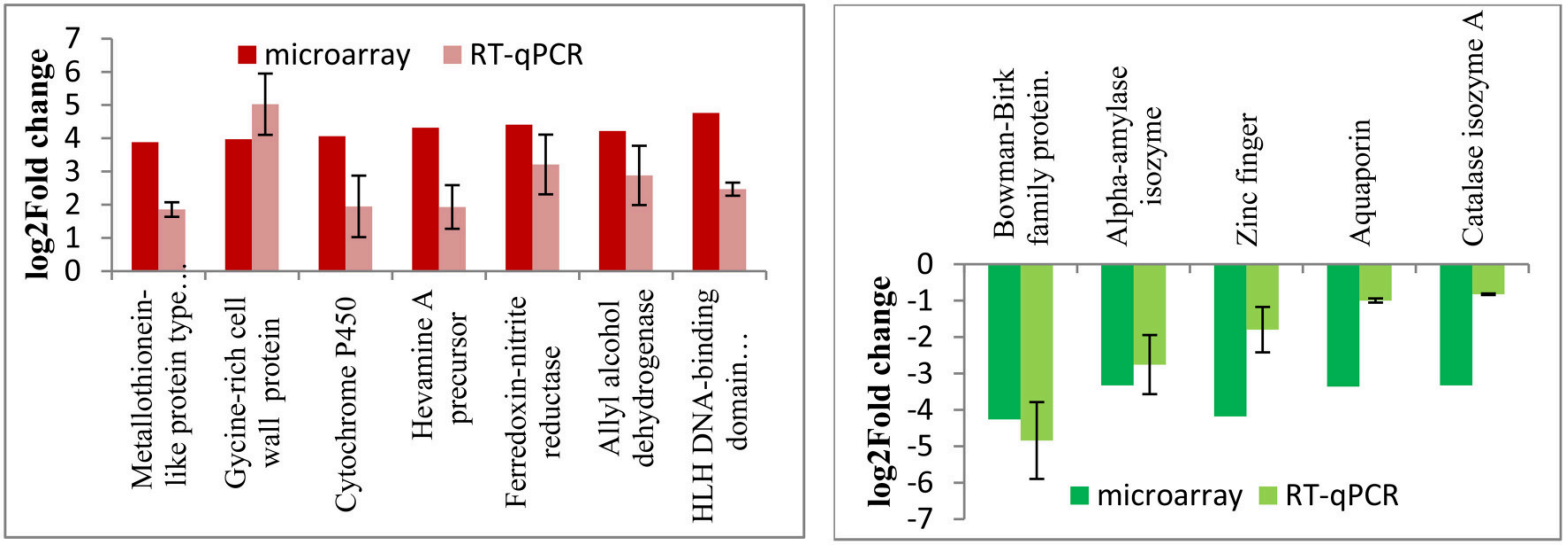
**qRT-PCR validation of RGA1-regulated genes identified as common to various abiotic stresses**. Out of the 1498 RGA1-regulated genes identified as common to multiple abiotic stresses on the microarray, 12 of the most up/down-regulated genes were validated by qRT-PCR. Their fold change data are shown based on averages of two biological replicates and two technical replicates of total RNA, along with microarray results. The left panel in red shows the up-regulated genes and the right panel in green shows the down-regulated genes.

In order to validate the stress-responsive genes identified from the microarray results, quantitative RT-PCR experiments were carried out using total RNAs isolated from the wild type and RGA1 mutant rice plants grown and harvested under similar conditions. Out of the 1498 RGA1-regulated genes identified as common to multiple abiotic stresses on the microarray, 12 of the most up/downregulated genes were validated by qRT-PCR. Their fold change data are shown in Figure 7 along with microarray results. The data clearly show the broad correspondence between the microarray data and RT-PCR results for both upregulated and downregulated sets of genes.

**Figure 8:**
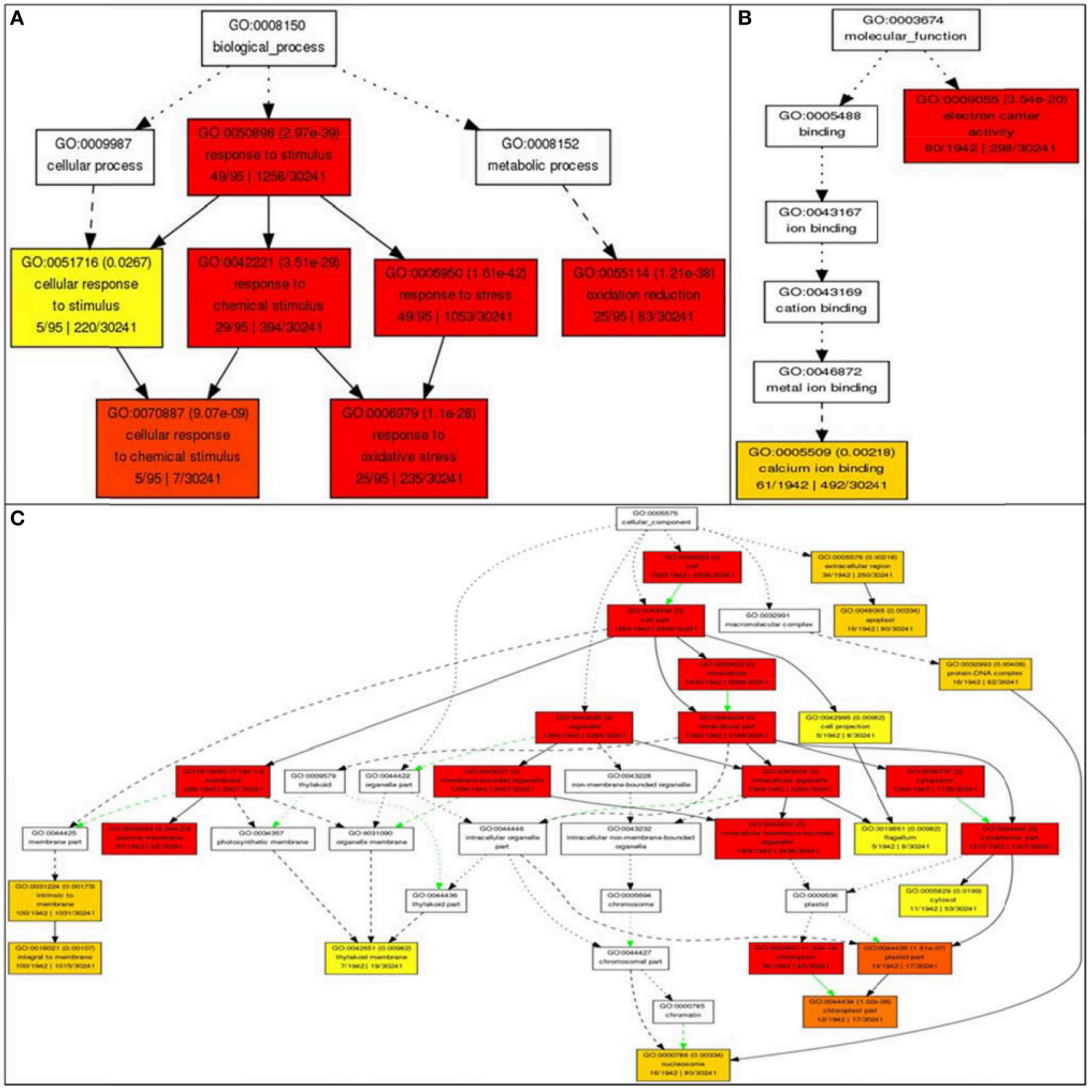
**Gene Ontology enrichment of RGA1-regulated, stress responsive genes from Rice DB**. The 1498 genes common to all four major abiotic stresses were subjected to GO enrichment using AgriGO with default settings. **(A)** Biological process categorization of the RGA1-regulated genes shared by salt, heat, cold, and drought stresses. **(B)** Molecular function categorization and **(C)** Subcellular localization of the RGA1-regulated genes shared by all four abiotic-stresses.

Gene ontology analysis of the core list of 1498 genes shared by all four stresses revealed 62 unique GO terms associated with molecular functions such as transporter activity, enzyme regulator activity, transferase activity, hydrolase activity, metabolic processes (carbon and protein), binding to nucleotides, carbohydrates, receptors and lipids, anatomical structure morphogenesis, flower development, and cell homeostasis (Supplementary Table 1). Further analysis using AGRIGO showed that many of these 1498 shared stress-responsive genes also share many GO terms of biological process, such as response to stimuli (GO: 0050896) with 49 genes out of the 95 genes (or 51%) accepted by AGRIGO for the query; 29 genes (30%) in response to chemical stimulus (GO: 0042221), 49 genes (51%) in response to stress (GO:0006950); 25 genes (26%) belong to oxidation reduction (GO:0055114); five genes (5%) belong to the category cellular response to chemical stimulus (GO:0070887), and 25 genes (26%) belong to response to oxidative stress (GO:0006979; Figure 8). This reveals the role of RGA1 in regulating a diverse range of processes related to stress response. GO terms of molecular function such as electron carrier activity had 80 genes (4%) and 61 genes (3%) in calcium ion binding out of a total of 1942 genes, indicating the role of RGA1 in their regulation. Its role also seems to be important in regulating the products of diverse cellular locations, such as etioplasts (130 genes), mitochondria (33 genes), plastid (16 genes), nucleus (15 genes), chloroplast (12 genes), and three genes each in endoplasmic reticulum, vacuole, and golgi apparatus (Figure 8).

### Mining for miRNAs targeting RGA1-regulated, stress responsive genes

Data mining for miRNAs at Rice DB using the GO terms of 1498 RGA1-regulated genes shared by all four stresses revealed that 63 of them could be targets of miRNAs. This indicates the role of RGA1 in post-transcriptional regulation of 63 target genes for the first time. They include 38 up-regulated genes and 25 down-regulated genes identified in the RGA1 mutant (Table 3).

**Table 3:** **miRNA targets among RGA1-regulated stress-responsive genes**.

**Gene identifier**	**miRNA_gene**	**Rap_description**	**GOID**
Os11g0119100	OsSBS99	Catalytic/hydrolase	GO:0008152
Os11g0115400	OsSBS97	Lipid transfer protein LPT IV	NA
Os01g0679600	OsSBS87	THAP domain-containing protein 4	GO:0003674
Os03g0206400	OsSBS73	Conserved hypothetical protein	GO:0003674
Os10g0181200	OsSBS68	Protein prenyltransferase	GO:0006139
Os08g0562600	OsSBS67ab	C2 calcium-dependent membrane targeting	GO:0003674
Os10g0391400	OsSBS58	Tify domain containing protein	GO:0006950
Os03g0818400	OsSBS53	40S ribosomal protein S23 (S12)	GO:0005840
Os05g0128200	OsSBS45	Hypothetical conserved gene	GO:0009058
Os04g0396800	OsSBS4	Serine carboxypeptidase K10B2.2	GO:0005618
Os03g0126000	OsSBS27	Phosphorybosylanthranilatetransferase 1	GO:0008152
Os08g0561700	osa-MIR552	Superoxide dismutase	GO:0008152
Os03g0738400	osa-MIR530-5p′	Serine hydroxymethyltransferase,)	GO:0005739
Os02g0761400	osa-MIR530-3p	ATPREP2	GO:0005576
Os08g0137400	osa-MIR530	Cupredoxin domain containing protein	GO:0003674
Os09g0428000	osa-MIR529b	Glycosyltransferase, family 2 domain protein	GO:0016740
Os01g0823600	osa-MIR444f.17	Conserved hypothetical protein	GO:0003674
Os02g0324400	osa-MIR444c-5p	FON2 SPARE1	GO:0005576
Os02g0274900	osa-MIR444bc.9	Metabolite transport protein csbC	GO:0006810
Os07g0583600	osa-MIR444bc.25	Chitin-inducible gibberellin-responsive protein	GO:0007165
Os03g0802500	osa-MIR444ad.2,e	ATPase, AAA-type, core domain	GO:0005783
Os08g0482700	osa-MIR444	Conserved hypothetical protein	GO:0003674
Os04g0459600	osa-MIR442	Mog1/PsbP, alpha/beta/alpha sandwich domain	NA
Os05g0414700	osa-MIR403	Brassinosteroid insensitive 1 receptor kinase 1	GO:0005102
Os05g0557700	osa-MIR399j	Conserved hypothetical protein	GO:0005575
Os01g0850700	osa-MIR397b′	Cupredoxin domain containing protein	GO:0009056
Os01g0121600	osa-MIR396c-3p	Conserved hypothetical protein	GO:0006810
Os01g0180800	osa-MIR396c	Heat shock protein Hsp70 family protein	GO:0005634
Os03g0225500	osa-MIR395f ′	Nucleoporin, Nup133/Nup155-	GO:0005515
Os05g0574500	osa-MIR395c′o′	GTP-binding nuclear protein Ran1B	GO:0005515
Os03g0195300	osa-MIR395	Low affinity sulfate transporter 3	GO:0016020
Os03g0559700	osa-MIR393ab′	Conserved hypothetical protein	NA
Os03g0388900	osa-MIR319a.2	Peptidase C14, caspase catalytic protein	GO:0019538
Os01g0323600	osa-MIR2055′	S-adenosylmethionine synthase 2	GO:0016020
Os03g0409100	osa-MIR1884b-3p	PUA-like domain domain protein	GO:0019538
Os08g0417000	osa-MIR1884b	2OG-Fe (II) oxygenase domain protein	GO:0008152
Os10g0462900	osa-MIR1879′	Mitochondrial chaperonin-60	GO:0005739
Os08g0512400	osa-MIR1879	Unknown function DUF296 domain protein	GO:0003677
Os07g0492000	osa-MIR1862	Nucleoside diphosphate kinase I (NDK I)	GO:0005576
Os01g0763200	osa-MIR1861d	Transcription factor PCF7 (Fragment)	GO:0005575
Os02g0830700	osa-MIR1860-5p	Leucine-rich repeat	GO:0005623
Os09g0530700	osa-MIR1860-3p	Hypothetical conserved gene	GO:0003674
Os02g0580900	osa-MIR1858b	TGF-beta receptor	NA
Os03g0667100	osa-MIR1851	BTB/POZ domain containing protein	GO:0006950
Os10g0510000	osa-MIR1850	Actin	GO:0009719
Os01g0720600	osa-MIR1847.7	Starch synthase IVa	GO:0016740
Os03g0101300	osa-MIR1847.10	Hexose transporter	GO:0005829
Os03g0821100	osa-MIR1846d-5p	Non-cell-autonomous HS cognate protein 70	GO:0016020
Os07g0684800	osa-MIR1846c-5p	NAM/CUC2-like protein	GO:0005634
Os08g0357000	osa-MIR172c	Wound responsive protein	GO:0006950
Os04g0442000	osa-MIR172b	Auxin response factor 2 (ARF1-BP)	GO:0009058
Os03g0784000	osa-MIR172ad	FAD dependent oxidoreductase family protein	GO:0008152
Os02g0662700	osa-MIR171i	Scl1 protein (Fragment)	GO:0008150
Os03g0696300	osa-MIR169no	Nuclear transcription factor Y subunit A-1	GO:0007275
Os03g0687000	osa-MIR168a	Predicted protein	GO:0016020
Os03g0640800	osa-MIR166m	(Homeodomain-leucine zipper protein 14)	GO:0003677
Os12g0147800	osa-MIR164d	Phytosulfokines 5 precursor	NA
Os05g0580000	osa-MIR162b	ADP-glucose pyrophosphorylase (EC 2.7.7.27)	GO:0009058
Os03g0140200	osa-MIR1441	Cytochrome P450 86A1	GO:0005623
Os02g0821200	osa-MIR1428	Ribosomal protein L28e domain protein	GO:0005840
Os01g0967800	osa-MIR1328	Conserved hypothetical protein	NA
Os10g0503800	osa-MIR1322	Remorin	GO:0005575
Os06g0195900	NA	NOG, C-terminal domain containing protein	GO:0016020

*Data mining at Rice DB using them are targets for miRNA regulation*.

## Discussion

Heterotrimeric G-protein subunits or their interacting partners have either been implicated in stress signal transduction or have been shown to respond to stress themselves (Urano et al., 2013). Experimental approaches, including genome-wide studies, were generally focused on the response to individual stresses or individual components of G-protein signaling. The role of the G-protein α subunit in individual abiotic stress responses has been in particular focus, in relation to heat/salt stress in pea (Misra et al., 2007) and salt stress in Arabidopsis (Colaneri et al., 2014), rice and maize (Urano et al., 2014), or indirectly in ABA signaling (Pandey et al., 2010; Alvarez et al., 2011) or oxidative stress (Booker et al., 2012). The expression of rice Gα subunit (RGA1) gene itself was reported to be up-regulated by salt, cold, and drought stresses, and down regulated by heat stress (Yadav et al., 2013). However, there are no comprehensive studies on the genome-wide involvement of any heterotrimeric G-protein subunit in all the main abiotic stresses in any plant, except Arabidopsis (Chakraborty et al., 2015a,b). Comprehensive functional genomic analyses are particularly lacking on the genome-wide role of RGA1 or other G-protein subunits in multiple abiotic stress responses in rice.

In view of our own recent findings reported elsewhere in this issue on the growing importance of G-protein signaling components in abiotic stress response in *Arabidopsis* (Chakraborty et al., 2015c), as well as the importance of abiotic stress in rice crop improvement, we sought to examine the abiotic stress component of our RGA1 transcriptome microarray data in detail. This was done by combining our experimental functional genomic data with *in silico* meta data analysis to answer the following questions: Does abiotic stress figure prominently in the genome-wide response to RGA1 null mutation in rice and if yes, what are the various genes involved and how are they distributed in terms of major individual abiotic-stresses or in terms of their differential regulation in the RGA1 mutant? How do they compare with the known genome-wide response of normal rice plants to various abiotic stresses? Can *in silico* transcriptome meta-data analyses provide adequate insights for integrative understanding on abiotic stress signaling components in rice as possible converging points for interventions?

Our microarray experiments under MIAME compliant conditions using the Japonica rice RGA1 mutant and wild type (GSE 20925 at NCBI GEO) revealed 2270 differentially expressed genes, out of which the stress responsive data set was identified and analyzed using three approaches: Gene Ontology terms, data mining from STIFDB, and meta-data analysis from Rice DB. Firstly, segregation using Gene Ontology terms yielded 94 genes corresponding to various abiotic stress categories, most of which belonged to less than 40 families (Table 1), indicating their regulation by RGA1. The fact that majority of these families showed similar patterns of up/down regulation indicates that their regulation by RGA1 is also uniform, while there are a few families such as those related to oxidative stress response that show differential regulation of their members in the RGA1 mutant. The uniform mode of up/down regulation of multiple members of the same family of stress-responsive genes reveals the inherently coordinated pattern of gene regulation in response to a stress signal (e.g., DREB), whereas the varied extent of that regulation reveals the fine tuning of the signal/response flux through a regulatory cascade. Such patterns of regulation may be amenable to deeper network analysis.

Secondly, data mining for genes specifically categorized as stress-responsive genes from Japonica rice at STIFDB yielded 626 genes, out which 106 genes belonging to various abiotic stresses—heat drought, salt cold, were RGA1-regulated (Figure 1). Together, these 106 abiotic stress-responsive genes constitute less than 5% of all the G-protein (RGA1) regulated genes. But they constitute a far higher proportion (17%) of the 626 abiotic stress-responsive genes, indicating the larger role for G-proteins in regulating them, even though mediating abiotic stress seems to be a smaller part of the genome-wide role of G-proteins. However, this difference may also be an artifact arising out of the relatively lesser coverage of 626 rice stress-responsive genes on the STIFDB, as compared to 3150 genes in *Arabidopsis*, as similar analysis on its GPA1 mutant produced more consistent ranking with cold>salt>drought (Chakraborty et al., 2015c).

Hierarchical clustering of the 106 RGA1-regulated, stress-responsive genes mined from STIFDB2.0 using Genevestigator revealed prominent clusters of cold and drought responsive genes (Figure 2), which were subjected to further analysis by biclustering using the same software. While hierarchical clustering helps in grouping genes with similar profiles across all abiotic stress conditions, Biclustering identifies groups of genes that exhibit similarity only in a subset of conditions such as cold or drought, irrespective of their expression profiles in other conditions. The regulation of genes identified as highly differentially regulated by biclustering in both cold and drought conditions was compared with the fold-change values obtained on our microarray (Figures 3, 4). This revealed that some of the genes follow similar pattern of regulation between the stress response in normal rice plants and the RGA1-response in mutants unexposed to stress. While these may indicate independent regulation, the remaining genes that follow opposite pattern of regulation could be due to the RGA1 mutation, suggesting that RGA1 may mediate the response of these genes to cold or drought stresses.

Thirdly, metadata analyses based on data mining at Rice DB using the 2270 genes we identified in the RGA1-transcriptome microarray revealed a much larger number of 1886 stress-related genes as differentially regulated in our RGA1 mutant. A Venn selection of the stress-responsive gene lists identified by all three approaches used in this study viz., gene ontology (94), STIFDB2.0 (106) and Rice DB (1886) revealed that the former two are largely subsets of the 1886 DEGs identified using Rice DB (Figure 5). Their Venn selections in terms of individual abiotic stress categories and by up/down regulation on our microarray (Figure 6) revealed 1498 genes as common to all four stresses, with fewer common genes in smaller combinations of stresses (Table 2). Out of them, 12 of the most up/down-regulated genes have been validated by qRT-PCR (Figure 7), confirming the broad trends of up/down regulated genes identified on the microarray. These include the well-known stress-responsive genes such as catalase and aquaporin. Among the individual stresses, the sheer number of RGA1-regulated genes that only respond to heat (and no other abiotic stress) is striking, and needs further analysis. Comparative microarray or RT-PCR profiling of the RGA1 mutant and wild type rice plants exposed to various abiotic stresses would reveal more details in this regard.

Gene Ontology enrichment of the 1498 RGA1-regulated genes shared by all four abiotic stresses using AGRIGO revealed their molecular functions, cellular localizations, and biological processes (Figure 8). In terms of processes, genes from the various abiotic stress signaling pathways such as calcium-dependent pathways, ABA dependent or independent pathways, and MAP kinase pathways, as well as various pathways involved in the production of osmoprotectants, heat shock proteins, metallothioneins, antioxidants etc., were found to be differentially regulated in the RGA1 mutant as elaborated below. Together, they clearly indicate the crucial role of G-protein alpha subunit signaling in transducing/mediating the response of rice to multiple abiotic stresses.

### Calcium-dependent pathways in G protein-mediated abiotic stress signaling

Calcium is a well-known second messenger in abiotic signal transduction and various calcium binding proteins such as calmodulins, calcineurin, CDPKs, and calcineurin B-like interacting protein kinases (CIPK) play an important role in calcium-dependent abiotic stress (Batistič and Kudla, 2012). The CBL proteins form a complex network with their target kinases CIPKs and regulate target gene expression (Das and Pandey, 2010). Some genes related to calcium signaling were shown to be involved in stress signaling in rice (Batistič and Kudla, 2012), and transgenic manipulation of some such genes has been shown to improve stress-tolerance in rice (Campo et al., 2014). In our study, calcium dependent protein kinase (Os02g0685900) is up regulated while Calmodulin-like protein CaML3 (Os11g0141400) is down regulated in the RGA1 mutant. Their further validation could help determine their potential as candidate genes for development of rice plants tolerant to multiple abiotic stresses.

### Map kinase pathways in G-protein-mediated abiotic stress signaling

Many MAPKs have been reported in rice for various abiotic stresses (Danquah et al., 2014). The MAPK gene OsMSRMK2 is highly induced by a variety of stresses including ABA, JA, SA, drought, and salt but not by cold (Danquah et al., 2014). In this study, for example, we found MAP kinase (Os03g0285800) to be up regulated. A receptor-like kinase, or *O. sativa* stress-induced protein kinase gene 1, which is known to be involved in drought and salt stress tolerance is also induced in the RGA1 mutant.

### ABA signaling in G-protein mediated abiotic stress response

ABA is involved in the regulation of many aspects of plant growth and development and also is the major hormone that controls plant responses to abiotic stresses (Danquah et al., 2014), especially drought stress. ABA is also one of the most studied hormones in relation to G-protein signaling (Zhao et al., 2010). We found a related gene encoding the SNF1-related protein kinase regulatory gamma subunit 1 (AKIN gamma1Os04g0382300) to be suppressed in the RGA1 mutant. Similarly, drought- responsive element binding protein (DREB) is a part of ABA-independent pathway, from which both DREB2 and CRT/DRE binding protein were up regulated in our data. Among the ABA signaling pathway genes, we also found that MYB expression was enhanced in the RGA1 mutants as compared with wild-type plants. Abscisic acid responsive element-binding factors belong to the ABA dependent pathway, of which AREB2 was up regulated in our data. Members of TF families that are involved in both ABA-independent (AP2/ERF and WRKY) and ABA dependent pathways are also involved in stress tolerance (Song et al., 2012).

### Transcription factors and miRNAs in G protein-mediated abiotic stress response

The expression of many stress responsive genes is mediated by transcription factors that bind to specific cis-elements in the promoters of their target genes. We found various transcription factors such as ADH1, OsNAC5, OsWRKY45, bZIP23/72 to be differentially regulated in our RGA1 mutant. Further characterization and validation of the transcription factors identified in our study may reveal their potential as candidate genes to engineer tolerance to various abiotic stresses in rice. At the post-transcriptional level, miRNAs are also known to play important regulatory roles in plant development and stress. miRNAs, such as miR168, miR171, and miR396, are regulated by abiotic stresses such as salinity, drought, and cold in rice (Mal et al., 2015). So far, no study has reported RGA1-responsive miRNAs involved in stress. In this study, we have mined 63 RGA1-regulated target genes for miRNAs that are also stress responsive. Further validation of their role in stress-response could reveal if they have any potential in crop improvement.

### Osmoprotectant genes, lea genes, heat shock proteins, and others

Several genes found to be differentially regulated in the RGA1 mutant belong to biosynthetic pathways of osmoprotectants such as polyamine, glycine-betaine, proline, and trehalose. Three genes (Os08g0445700, Os02g0661100, and Os01g0749400) involved in the trehalose synthesis pathway were up regulated in the RGA1 mutant. A major pathway that is significantly down regulated is the betanidin degradation pathway with 21 genes being down regulated. Two genes (Os03g0738400, Os12g0409000) from the glycine betaine synthesis were also up regulated in the RGA1 mutant. These genes are known to be involved in various stress responses such as increased submergence tolerance, drought, and cold resistance (Marco et al., 2015).

Heat shock proteins and molecular chaperone proteins like metallothionein proteins are involved in heat and drought tolerance. Their genes were highly up regulated in the RGA1 mutant with fold changes up to three and validated by qRT-PCR (Figure 7). Late embryogenesis abundant (LEA protein) genes are known to help in drought and salinity tolerance (Mondini and Pagnotta, 2015). In our study, Lea14-A was up-regulated in the RGA1 mutant, indicating its potential importance in rice stress. Similarly, among the hormone regulatory genes, we found RGA1-regulation of IPT and ABA hydroxylase, which delay senescence and yield under drought and reduce sterility under cold stress. Oxidative stress related genes such as Glutathione S-transferase and superoxide dismutase genes are involved in salt and cold stress (Marco et al., 2015). In our study, superoxide dismutase was down regulated in the RGA1 mutant, indicating the important role of G-protein alpha subunit in SOD-mediated regulation of oxidative stress. Genes encoding proton pumps, antiporters, and ion transporters like vacuolar Na+/H+ antiporter and aquaporins are also known to enhance salt and cold tolerance. Our data shows that aquaporins are down regulated 3.36 times in the RGA1 mutant and is validated by qRT-PCR (Figure 7).

## Conclusion

Overall, our results clearly indicate the potentially crucial role of the G-protein α subunit (RGA1) in regulating the response of the rice plant to multiple abiotic stresses for further experimental validation. The 1886 RGA1-regulated and stress-responsive genes we mined in this study may represent only a subset of overall-stress responsive genes in rice, but they do constitute a G-protein (RGA1)-regulated subset that was never described in any plant so far, except in Arabidopsis elsewhere in this issue (Chakraborty et al., 2015c). The fact that as many as 1498 RGA1-regulated, stress-responsive genes are common to the four abiotic stresses (drought, salt, heat, cold), and that relatively fewer genes are uniquely regulated by RGA1 in response to individual stresses indicates that RGA1-signaling could be a converging point for the regulation of multiple abiotic stress responses. Its experimental validation, as well as that of the exceptionally large number of 111 unique genes regulated by RGA1 in heat stress (unshared with the other three stresses) could offer glimpses into the commonalities and differences in heat stress signaling vis-à-vis other stresses.

### Conflict of interest statement

The authors declare that the research was conducted in the absence of any commercial or financial relationships that could be construed as a potential conflict of interest.
