# Supplementary material for: Microarray Analysis of Rice d1 (RGA1) Mutant Reveals the Potential Role of G-Protein Alpha Subunit in Regulating Multiple Abiotic Stresses Such as Drought, Salinity, Heat, and Cold
Source: Front Plant Sci. 2016 Jan 28;7:11. doi: 10.3389/fpls.2016.00011 (PMC4729950; doi:10.3389/fpls.2016.00011)
Supplement: Supplementary file 1 [file DataSheet1.docx]

**Supplementary Table 1: Gene ontology of the core list of stress responsive genes shared by 4 abiotic stresses.**

| **IDENTIFIER** | **LOCID** | **GOID** | **GO_DEFINITION** |
| --- | --- | --- | --- |
| Os06g0195900 | LOC_Os06g09570.1 | GO:0016020 | Membrane |
| Os04g0476800 | LOC_Os04g40090.1 | GO:0005488 | Binding |
| Os01g0149800 | LOC_Os01g05650.1 | GO:0019725 | cellular homeostasis |
| Os04g0442000 | LOC_Os04g36054.1 | GO:0009058 | biosynthetic process |
| Os01g0822900 | LOC_Os01g60740.1 | GO:0005623 | Cell |
| Os02g0721700 | LOC_Os02g48980.1 | GO:0003674 | molecular_function |
| Os04g0493100 | LOC_Os04g41570.1 | GO:0005634 | Nucleus |
| Os05g0322900 | LOC_Os05g25770.1 | GO:0008150 | biological_process |
| Os11g0174000 | LOC_Os11g07280.1 | GO:0006810 | Transport |
| Os09g0535000 | LOC_Os09g36450.1 | GO:0000003 | Reproduction |
| Os05g0421600 | LOC_Os05g34830.1 | GO:0006950 | response to stress |
| Os03g0225500 | LOC_Os03g12450.1 | GO:0005515 | protein binding |
| Os07g0694700 | LOC_Os07g49400.1 | GO:0003824 | catalytic activity |
| Os08g0559600 | LOC_Os08g44530.1 | GO:0007275 | multicellular organismal development |
| Os05g0556900 | LOC_Os05g48310.1 | GO:0005840 | Ribosome |
| Os05g0566400 | LOC_Os05g49140.1 | GO:0007165 | signal transduction |
| Os03g0149200 | LOC_Os03g05520.1 | GO:0005783 | endoplasmic reticulum |
| Os05g0560600 | LOC_Os05g48690.1 | GO:0005575 | cellular_component |
| Os07g0475900 | LOC_Os07g29330.1 | GO:0016301 | kinase activity |
| Os06g0107700 | LOC_Os06g01850.1 | GO:0005576 | extracellular region |
| Os01g0521400 | LOC_Os01g33784.1 | GO:0008152 | metabolic process |
| Os01g0769700 | LOC_Os01g56330.1 | GO:0009856 | Pollination |
| Os10g0462900 | LOC_Os10g32550.1 | GO:0005739 | Mitochondrion |
| Os02g0661100 | LOC_Os02g44230.1 | GO:0016787 | hydrolase activity |
| Os05g0498400 | LOC_Os05g41900.1 | GO:0006412 | Translation |
| Os02g0266800 | LOC_Os02g16680.1 | GO:0003677 | DNA binding |
| Os01g0246400 | LOC_Os01g14410.1 | GO:0009628 | response to abiotic stimulus |
| Os09g0568400 | LOC_Os09g39500.1 | GO:0005730 | Nucleolus |
| Os05g0176100 | LOC_Os05g08370.1 | GO:0016740 | transferase activity |
| Os01g0744000 | LOC_Os01g54080.1 | GO:0009987 | cellular process |
| Os06g0116600 | LOC_Os06g02600.1 | GO:0016043 | cellular component organization |
| Os01g0383700 | LOC_Os01g28680.1 | GO:0005622 | Intracellular |
| Os04g0463500 | LOC_Os04g38950.1 | GO:0009791 | post-embryonic development |
| Os01g0720400 | LOC_Os01g52230.1 | GO:0009991 | response to extracellular stimulus |
| Os04g0151800 | LOC_Os04g06770.1 | GO:0003723 | RNA binding |
| Os01g0227100 | LOC_Os01g12710.1 | GO:0019538 | protein metabolic process |
| Os12g0593000 | LOC_Os12g40190.1 | GO:0009653 | anatomical structure morphogenesis |
| Os04g0517100 | LOC_Os04g43680.1 | GO:0009719 | response to endogenous stimulus |
| Os04g0671900 | LOC_Os04g57610.1 | GO:0009908 | flower development |
| Os03g0278300 | LOC_Os03g17010.1 | GO:0000166 | nucleotide binding |
| Os07g0600700 | LOC_Os07g40940.1 | GO:0030246 | carbohydrate binding |
| Os07g0574800 | LOC_Os07g38730.1 | GO:0005856 | Cytoskeleton |
| Os07g0143000 | LOC_Os07g05000.1 | GO:0009536 | Plastid |
| Os05g0495700 | LOC_Os05g41590.1 | GO:0005975 | carbohydrate metabolic process |
| Os07g0658300 | LOC_Os07g46450.1 | GO:0030234 | enzyme regulator activity |
| Os01g0948500 | LOC_Os01g72000.1 | GO:0006464 | protein modification process |
| Os01g0565900 | LOC_Os01g38510.1 | GO:0005215 | transporter activity |
| Os05g0585400 | LOC_Os05g50800.1 | GO:0005737 | Cytoplasm |
| Os05g0414700 | LOC_Os05g34270.1 | GO:0005102 | receptor binding |
| Os08g0230200 | LOC_Os08g13350.1 | GO:0005886 | plasma membrane |
| Os02g0285800 | LOC_Os02g18450.1 | GO:0009056 | catabolic process |
| Os03g0385400 | LOC_Os03g26820.1 | GO:0008289 | lipid binding |
| Os11g0543100 | LOC_Os11g34080.1 | GO:0008219 | cell death |
| Os01g0276000 | LOC_Os01g16890.1 | GO:0005829 | Cytosol |
| Os09g0120800 | LOC_Os09g03310.1 | GO:0006139 | Nucleo-base/side/tide and nucleic acid metabolism |
| Os04g0396800 | LOC_Os04g32540.1 | GO:0005618 | cell wall |
| Os07g0577600 | LOC_Os07g38960.1 | GO:0006091 | generation of precursor metabolites and energy |
| Os02g0625000 | LOC_Os02g41550.1 | GO:0004872 | receptor activity |
| Os05g0231700 | LOC_Os05g14240.1 | GO:0005773 | Vacuole |
| Os10g0522000 | LOC_Os10g37770.1 | GO:0005794 | Golgi apparatus |
| Os05g0360400 | LOC_Os05g29710.1 | GO:0009607 | response to biotic stimulus |
| Os04g0554200 | LOC_Os04g46780.1 | GO:0005198 | structural molecule activity |

**Supplementary Table 2:** The primer sequences used for Real time PCR validation. The up regulated and down regulated gene IDs are shown in red and green font respectively.

| **IDENTIFIER** | **Primer Sequence** |
| --- | --- |
| Os01g0149800 | OsqMtpupF GTGCTGCGGAGGAAACT  OsqMtepupR CATGATGACAGTCTGGGTAGTG |
| Os10g0450900 | OsqGspupF ACTACCCAGCTCACTGAAGTA  OsqGspupR ACCAATGCTAAGGAGGACAAG |
| Os02g0703600 | OsqCycupF CGAACGGTGGATTCCGTATTAG  OsqCycupR TCATCAACTCCTCTGCTCCT |
| Os01g0860500 | OsqChiupF GTGAAGAAGTCTCCCAAGTACG  OsqChiupR TAGCTCAAACGCTGGACTTC |
| Os01g0357100 | OsqFnrupF GACGAGGAGTAGGAACACAGA  OsqFnrupR CCATGGGCGAAGGCATATT |
| Os04g0497000 | OsqAdhupF CTCACATCGCAGAGCAATCT  OsqAdhupR GCTGCCATCTTTGGTCTCT |
| Os01g0149800 | OsqHlhupF TGGCCACCTACTTTACCTAGTC  OsqHlhupR AACCACCTCCATCACGTAGT |
| Os01g0124400 | OsqBbpdwF CATCTGCGAGGACATCTACTG  OsqBbpdwR GTCGGCGGGTTCATCTT |
| Os08g0473900 | OsqAAPdwF GGAACCTGAAGCAGGAGATAAC  OsqAAPdwR CGATCTTCACCATGACCTTCTC |
| Os10g0544600 | OsqZfdwF CTCGTGAAGATGGAGAGAGAATG  OsqZfdwR ACCTCAACGCAACAGAGATAC |
| Os07g0448800 | OsqAquadwF GCGTTCTACCACCAGTACATC  OsqAquadwR ACGCCTTCCAGCCATTATC |
| Os02g0115700 | OsqCatdwF CGGATAGACAGGAGAGGTTCAT  OsqCatdwR ATCACACTGGGAGAGGTAGTT |
